# Fluorescein sodium in the surgical treatment of pleomorphic xanthoastrocytomas: Results from a retrospective study

**DOI:** 10.3389/fonc.2022.1009796

**Published:** 2022-11-14

**Authors:** Jacopo Falco, Morgan Broggi, Ignazio G. Vetrano, Emanuele Rubiu, Marco Schiariti, Francesco Restelli, Elio Mazzapicchi, Giulio Bonomo, Emanuele La Corte, Paolo Ferroli, Francesco Acerbi

**Affiliations:** ^1^ Department of Neurosurgery, Fondazione IRCCS Istituto Neurologico Carlo Besta, Milan, Italy; ^2^ Department of Biomedical Sciences for Health, Università degli Studi di Milano, Milan, Italy

**Keywords:** central nervous system tumors, neuro-oncology, yellow 560 filter, fluorescence-guided neurosurgery, pleomorphic xanthoastrocytoma (PXA), sodium fluorescein (SF)

## Abstract

**Objective:**

Pleomorphic xanthoastrocytoma (PXA) is a rare brain tumor, most commonly affecting children and young adults. Surgical resection represents the mainstay of treatment, and extent of resection is associated with improved survival. In this study, we analyzed the role of sodium fluorescein (SF) in improving intraoperative visualization easing resection.

**Methods:**

Surgical database of FLUOCERTUM study (Besta Institute, Milan, Italy) was retrospectively reviewed to find pleomorphic xanthoastrocytomas and anaplastic xanthoastrocytomas, according to WHO-2016/2021 classification, surgically removed by a fluorescein-guided technique from March 2016 to February 2022. SF was intravenously injected (5mg/kg) immediately after induction of general anesthesia. Tumors were removed using a microsurgical technique with the YELLOW 560 filter (*Carl Zeiss Meditec, Oberkochen, Germany*).

**Results:**

Twelve patients (7 males and 5 females; 3 pediatric patients, mean age 10 years, range 5 to 13 years and 9 adult patients, mean age 50.6 years, range 35 to 63 years) underwent fluorescein-guided surgery. No side effects related to SF occurred. In all tumors, contrast enhancement on preoperative MRI correlated with intense, heterogeneous yellow fluorescence with bright fluorescent cystic fluid. Fluorescein was considered helpful in distinguishing tumors from viable tissue in all cases. Gross total resection was achieved in 8 cases (66.7%); in 4 cases, otherwise, the resection was subtotal with fluorescent residual spots to avoid neurological worsening (33.3%).

**Conclusions:**

The use of SF is a valuable method for safe fluorescence-guided tumor resection. Our data documented a positive effect of fluorescein-guided surgery on intraoperative visualization, suggesting a probable role in improving the extent of resection during yellow surgery of PXA.

## Introduction

Pleomorphic xanthoastrocytoma (PXA) is a rare, low-grade glial tumor, commonly affecting young adults and children (1); no gender predilection was reported. According to the 2016 (2) and 2021 (3) World Health Organization (WHO) tumors classification, PXA are classified as grade II/2 or III/3 tumors; the latter, previously designated as pleomorphic xanthoastrocytoma with anaplastic features, are characterized by a mitotic index ≥ 5/10 high-power fields and are currently considered anaplastic gliomas. According to 2016 classification, pleomorphic xanthoastrocytomas and anaplastic pleomorphic xanthoastrocytomas were considered two different entities; conversely, according to WHO CNS5 version (3), PXA is a single tumor type, anaplastic has been excluded as a modifier term in favor of a grading diagnosis relative to the tumor type (i.e. grade 2 or 3) to provide more flexibility in categorization and to emphasize biological similarities within tumor types rather than approximate clinical behavior. Pleomorphic xanthoastrocytomas have a relatively favorable prognosis when compared with diffusely infiltrative astrocytomas (4).

PXA presented at MRI (5) with a heterogeneous contrast enhancement due to blood brain barrier (BBB) disruption, with peritumoral infiltrative/vasogenic brain edema; these tumors sometimes consist of solid and cystic components: the cystic fluid is generally isointense as compared with cerebrospinal fluid. Leptomeningeal enhancement is seen in a subset of patients.

Among circumscribed gliomas, PXA can present some infiltrative features: indeed, it can be difficult to distinguish between circumscribed and diffuse astrocytic gliomas. They can look very similar to epithelioid glioblastomas, especially for grade 3 PXA. Genomic methylation profiling (BRAF V600E, CDKN2A/B) is particularly helpful in such circumstances (6), as many tumors thought to be epithelioid glioblastomas actually map either to PXA or pediatric high-grade glioma.

Surgical resection is considered the cornerstone of therapy (1, 4, 7, 8); furthermore, recent reports suggest a prolonged survival in patient treated with a gross total resection (GTR). The role of adjuvant treatment in grade II/2 tumors has not been agreed upon and clinical practice varies widely; otherwise, patients affected by grade III/3 astrocytomas are generally scheduled for adjuvant Stupp protocol. Patients are usually followed up by regular imaging, as re-resection is advised at recurrence.

Sodium fluorescein (SF) is a dye that, when intravenously injected, has the peculiar characteristic to accumulate in cerebral areas presenting a damage of the BBB (9, 10). The use of a dedicated filter in the surgical microscope, with specific wavelength for fluorescein (540-690nm), allows to improve the tumor-brain discrimination intraoperatively, reducing also the dosage needed to obtain this effect (11–13). In particular, this has been shown to be associated to a better tumor identification and resection in different tumors of the central nervous system (CNS) such as high-grade gliomas (14, 15), gangliogliomas (16), cerebral metastasis (17), and primary CNS lymphomas (18), both in adult and pediatric population (19).

In July 2015, based upon preliminary scientific results from different studies published in the international literature (20, 21), including a prospective phase II trial from our group, the Italian Medicine Agency (AIFA) has extended the indications for the utilization of fluorescein molecule (https://www.gazzettaufficiale.it/atto/serie_generale/caricaDettaglioAtto/originario;jsessionid=izVcTOmnjOzfNRjjw56kAA:.ntc-as2-guri2b?atto.dataPubblicazioneGazzetta=2015-07-22&atto.codiceRedazionale=15A05620&elenco30giorni=false). According to this determination, the intravenous (i.v.) injection of SF as a neurosurgical tracer during oncological procedures for aggressive tumors of the CNS is approved and its cost is totally reimbursed by the Italian National Health System (15). A low dose (5 mg/kg) of fluorescein is i.v. administrated at the end of patient intubation (around one hour before dural opening). Since our first experience in 2011 in HGG resection (20) by means of YELLOW 560 filter (*Carl Zeiss Meditec, Oberkochen, Germany*), we have used the low dosage of 5 mg/kg for our subsequent studies and surgical procedures; this protocol has standardized SF usage independently from the characteristics of the patients or of the tumors.

In March 2016, the authors started a new prospective observational study, called FLUOCERTUM (FLUOrescein in CERebral TUMors), regarding the use of SF as a fluorescent intra-operative tracer in patients with suspected aggressive tumors of the CNS (15).

Due to the characteristics MRI contrast uptake in PXA, the use of SF as a fluorescent tracer could allow a better intraoperative discrimination of the tumoral tissue even in this tumor subtype, with beneficial effect during the surgical resection (22). The foundation of our study lays upon the intrinsic staining potential of fluorescein and the radiological features of PXA, characterized by an intense contrast-enhanced pattern (5). Since fluorescein molecule presents a slightly lower molecular weight than that of the contrast medium, the rational of its utilization is to selectively stain the contrast-enhanced areas of PXA to improve the intraoperative visualization of the tumor and its borders, better delineating even the components where contrast enhancement was not prominent (23). The principal aim of this paper consists in showing the advantages and the valuable role of fluorescein in visualization and resection of pleomorphic xanthoastrocytomas in our case series.

## Methods

### Patients and inclusion criteria

In this study, we retrospectively reviewed the database of the prospective observational FLUOCERTUM study, started in March 2016 and approved by the Institutional Review Board, to identify the cohort of patients affected by pleomorphic xanthoastrocytoma until February 2022. The FLUOCERTUM inclusion criteria were as follows: patients of both genders, at any age; patients with suspected aggressive lesions of the CNS, as suggested by preoperative MRI or CT with i.v. contrast agent administration. The exclusion criteria were: impossibility to give consent due to cognitive deficits or language disorders; known allergy to contrast agents or history of previous anaphylactic shocks; known severe previous adverse reactions to SF; acute myocardial infarction or stroke in the last 90 days; severe organ failure; women in their first trimester of pregnancy or lactation. The retrospective case series revision aims to identify all those consecutive patients scheduled for fluorescein-guided surgery with a histopathologically confirmed PXA according to 2016/2021 WHO CNS5 tumors classification.

### Clinical and radiological management

Preoperative assessment included physical and neurological examination (Karnofsky Performance Status [KPS] (24) in adult patients and Lansky Play-Performance status [LPS] (25) in pediatric ones), laboratory tests results and contrast-enhanced MRI for neuronavigation. In preoperative MRI, patients were categorized based on preoperative contrast enhancement characteristics. To evaluate the extent of resection (EOR), a volumetric MRI examination was performed for each patient within 72 hours after surgery; in particular, to calculate the residual pathological volume, the hyperintense alterations in volumetric basal T1 acquisitions were subtracted from the volume of hyperintense tissue in post-contrast volumetric T1 images, to avoid the incidental inclusion of blood or blood product (26). The EOR was calculated as a percentage of tumor resection based on early contrast-enhanced postoperative MRI; according to the entity of resection, we distinguished four main categories: GTR (EOR 100%), sub-total resection (STR, with an EOR of 90–100%), partial resection (PR, 30–90%) and biopsy. The postoperative clinical evaluation included a standard neurological examination as above as well as laboratory test (kidney function) and exclusion of occurrence of any side effect related to fluorescein injection. Clinical and neuroradiological long-term follow-up was performed for postoperative period as part of normal clinical practice, including telephonic interview.

### Surgical protocol

The standardized surgical protocol of fluorescein-guided technique, as already described in previous papers (11, 12, 15, 20, 21), is based on i.v. SF (*Monico S.p.A., Venice, Italy*) injection at standard dose of 5mg/kg, by a central or peripheral venous line, immediately upon completion of the induction of general anesthesia. Surgery was performed with the aid of a surgical microscope equipped with an integrated fluorescent filter tailored to the excitation and emission wavelength of sodium fluorescein (YELLOW 560 – *Pentero 900* and *Kinevo; Carl Zeiss Meditec, Oberkochen, Germany*). Surgical procedures were executed by different surgeons with the same philosophy regarding the importance and evaluation of SF and with the principle of the maximal safe resection. During resection, the microscope could be switched alternatively from fluorescent to white-light illumination; neuronavigation, intraoperative contrast enhanced ultrasounds or other tools could be used according to the surgeon’s preference. In tumors located adjacent to eloquent areas, intraoperative neurophysiological monitoring was used. Tumors were removed in an inside-out fashion until all fluorescent tissue was removed, as considered feasible by the surgeon.

### Intraoperative fluorescence characteristics and side effects

Fluorescence intensity was graded by the surgeon as intense, moderate, slight or absent; surgeons were also asked to classify the use of SF per each procedure as useful, useless or not essential to achieve surgical aims. Furthermore, medical reports were evaluated for any possible adverse effect or allergic reaction to fluorescein administration.

### Statistical analysis

The heterogeneous sample was described by means of the usual descriptive statistics: mean, median and standard deviation for continuous variables and proportions for categorical ones. PRISM software for Macintosh was used for the statistical analysis.

## Results

### Patients population

We enrolled 12 patients (7 males and 5 females; 3 pediatric patients, mean age 10 years, range 5 to 13 years and 9 adult patients, mean age 50.6 years, range 35 to 63 years) affected by pleomorphic xanthoastrocytomas (**Table 1**).

**Table 1:** Characteristics of the patients and main results.

Nr.	Age	Gender	Clinical presentation	KPS/LPS pre-op	Tumor location	Tumor enhancement	Fluorescence intensity	Intra-op side effects	Surgeon’s opinion	Residual fluorescence (Y/N - explanation)	EOR (GTR, intended STR, unintended STR, PR-debulking, biopsy)	Histology	KPS/LPS post-op	FU (Months)	FU (Radiological)	FU (KPS/LPS)
**1**	5	F	visual deficit	60	left temporo-parieto-occipital	heterogeneous and intense with cystic components	moderate	no	useful	N	GTR	anaplastic pleomorphic xanthoastrocytoma (WHO III)	70	55	tumor free	80
**2**	37	M	seizure	90	right temporal	heterogeneous and intense	intense	no	useful	N	GTR	pleomorphic xanthoastrocytoma (WHO II)	90	52	tumor free	100
**3**	12	F	seizure	90	left temporal	homogeneous and intense	intense	no	useful	N	GTR	pleomorphic xanthoastrocytoma (WHO II)	90	43	tumor free	90
**4**	51	F	worsening headache	100	left occipital	heterogeneous and intense with cystic components	intense (bright cystic fluid)	no	useful	N	GTR	pleomorphic xanthoastrocytoma (WHO II)	100	38	tumor free	100
**5**	41	M	left hemiplegia, headache	60	left fronto-temporo-insular	peripheral with central necrosis	intense	no	useful	Y – fluorescent tissue adherent to insular branches of MCA and to CST	iSTR	anaplastic pleomorphic xanthoastrocytoma (WHO III)	50	20	progression disease (–> CHT)	70
**6**	57	M	headache	80	left fronto-mesial, lateral ventricle	homogeneous and intense	intense	no	useful	N	GTR	anaplastic pleomorphic xanthoastrocytoma (WHO III)	70	31	tumor free	90
**7**	63	F	confusion	80	left temporal	peripheral with central necrosis	intense	no	useful	N	GTR	anaplastic pleomorphic xanthoastrocytoma (WHO III)	70	25	progression disease (–> palliative CHT)	30
**8**	54	F	right hand motor impairment	80	left frontal	heterogeneous and intense	intense	no	useful	N	GTR	anaplastic pleomorphic xanthoastrocytoma (WHO III)	70	10	progression disease (–> CHT)	80
**9**	57	M	right hemiparesis, disphasya, confusion	60	left temporo-insular	intense enhancement of the solid component with a large cyst	intense	no	useful	Y – fluorescent tissue infiltrating MCA perforators and CST	iSTR	anaplastic pleomorphic xanthoastrocytoma (WHO III)	60	5	progression disease (–> CHT)	80
**10**	35	M	headache	80	left peritrigonal	heterogeneous and intense with cystic components	intense fluorescence	no	useful	Y – fluorescent tissue adherent to CST and to arcuate fascicle	planned iSTR	pleomorphic xanthoastrocytoma (WHO II)	80	5	stable tumor	90
**11**	60	M	right arm and leg motor deficit	80	left front-parietal	heterogeneous and faint	intense fluorescence	no	useful	Y – fluorescent tissue adherent to CST	planned iSTR	pleomorphic xanthoastrocytoma (WHO 3)	60	3	progression disease (–> palliative CHT)	0
**12**	13	M	headache	100	left frontal	intense enhancement of the solid component with a large cyst	moderate (bright cystic fluid)	no	useful	N	GTR	pleomorphic xanthoastrocytoma (WHO 2)	100	2	tumor free	100

All patients presented with variable contrast enhancement pattern on the preoperative MRI: predominantly, PXA showed a heterogeneous enhancement (6/12 – 50%) with different degree of intensity and occasional presence of cystic components; in 2 cases (16.7%), the tumor presented a homogeneous and intense contrast enhancement whereas in other 2 cases (16.7%) the contrast uptake resulted peripheral and heterogeneous with central cystic-necrotic areas. Finally, 2 PXA (16.7%) appeared characterized by an intense enhancement of the solid, mural component with a large, satellite cyst (**Table 1**).

Most patients underwent surgery until 2021; 4 out of 12 cases (33.3%) resulted pleomorphic xanthoastrocytoma (grade II, WHO 2016), 6 of them (50.0%) were diagnosed anaplastic pleomorphic xanthoastrocytoma whereas 1 patient (8.3%) was affected by pleomorphic xanthoastrocytoma (grade 2, WHO CNS5 2021) and another (8.3%) by pleomorphic xanthoastrocytoma (grade 3, WHO CNS5 2021). Data on the clinical condition at admission, discharge, and follow-up and intraoperative findings were available for all patients (**Table 1**).

### Intraoperative fluorescence characteristics and surgeon’s opinion

**Figure 1:**
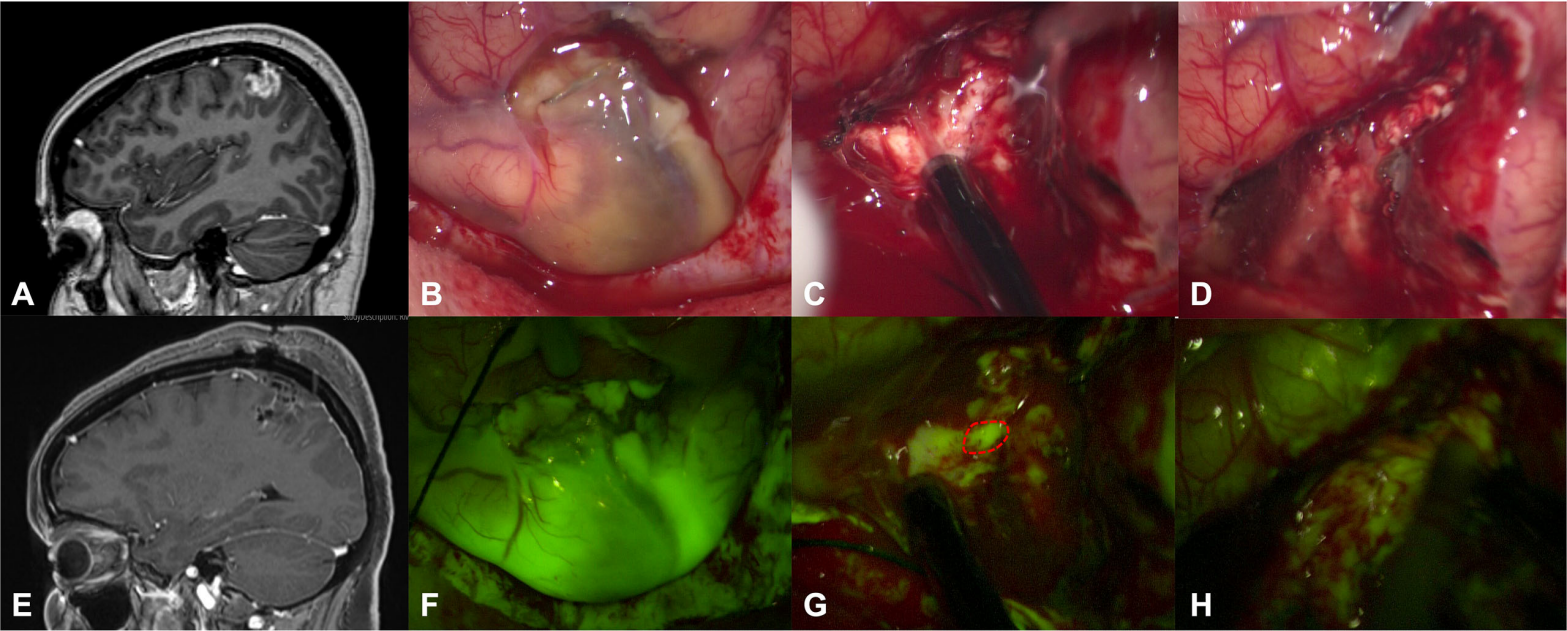
Preoperative T1 with contrast sagittal scan **(A)** in a 54-year-old female (patient n.8) shows a left frontal anaplastic pleomorphic xanthoastrocytoma with a gross-total resection, as detectable by postoperative post-contrast T1 MRI in **(E)**. After dural opening, it was possible to appreciate a greyish and pale lesion **(B)** that was better highlighted after activation of YELLOW 560 filter **(F)**. The tumor was removed with an inside-out fashion with the aid of a monopolar suction probe, showing a bleeding, friable tissue **(C)**. During surgical resection, fluorescein helps the surgeon to identify residual pathological tissue (dotted lines in **G**), not clearly visible under white light **(C)**. At the end of resection **(D, H)**, no more fluorescent/pathological areas were detectable with the YELLOW 560 filter activated or with white light illumination.

Intense fluorescent staining was reported in 10/12 cases (83.3%) whereas a moderate fluorescence enhancement was detected in 2 patients (16.7%). In 2 multicystic tumors, independently from specific fluorescein enhancement, we observed a bright fluorescent cystic fluid (**Table 1**). Five patients of our series (41.7%) were taking presurgical corticosteroid as a mild anti-edema (dexamethasone 4-8mg/day) and as a symptomatic relief; according to our case analysis, preoperative corticosteroid therapy did not affect fluorescence characteristics, as subjectively judicable by the surgeons. In all cases, intraoperative fluorescence was deemed helpful (**Table 1**) in achieving GTR by means of a better delineation of the borders of the tumor tissue from the health parenchyma, as compared to the conventional microsurgical technique using white-light illumination (**Figure 1**). Intraoperative fluorescence corresponded to preoperative MRI documented contrast enhancement. No technical difficulties regarding the use of the microscope filter nor switching between white and yellow light were encountered during the surgical resections.

### Extent of resection

GTR was achieved in 8 cases out of 12 patients scheduled for tumor removal (66.7%); in 2 cases (16.7%) the resection was planned as subtotal (planned STR) due to the volume of the lesion and the relationship with eloquent areas whereas in other 2 patients (16.7%), an intended STR was the consequence of the intraoperative identification of fluorescent residual spots in the context of eloquent tissue identified by means of intraoperative neurophysiological monitoring and due to tenacious adherences with MCA branches, in order to avoid neurological worsening (**Table 1**).

### Side effects and outcome

No adverse drug reaction related to SF injection was reported in this cohort of patients; the only remarkable and visible effect was the transient yellowish staining of urine which disappeared in about 24 hours.

At baseline, 9/12 (75%) of patients had KPS/LPS scores of 80-100, indicating good clinical and neurological conditions. Surgical morbidity led to a postoperative mild decline of KPS/LPS score in 5 patients; 6 patients were discharged with an unchanged KPS/LPS score whereas 1 patient presented a clinical improvement by means of surgical treatment. At discharge, 5/12 (41.7%) of patients had KPS/LPS scores of 80-100 (**Table 1**).

In addition to minor neurological complications related to the surgical manipulation of the specific eloquent structures, in 1 patient (n.5) a hemorrhagic complication requiring a re-craniotomy occurred (extradural hematoma); no significant neurological impairment derived from this emergency condition. Two patients with periventricular tumoral localization (n.6 and n.10) developed, in the postoperative course, a subacute obstructive hydrocephalus which required surgical management through positioning of a ventriculoperitoneal shunt.

The follow-up period ranged between 2 and 55 months, with a median follow-up of 24.1 months.

Tumor grading significantly influenced patient outcome; indeed, anaplastic PXA were characterized by a worst prognosis. Grade II/2 tumors were followed-up both clinically and radiologically whereas grade III/3 tumors were scheduled for Stupp protocol; no target or other pilot therapy was administered as a first line: B-Raf-inhibitor (Vemurafenib) was purposed in case of progression disease (27). Follow-up data were available for all patients.

Regarding grade II/2 PXA, all 5 patients were neuroradiologically stable (tumor free or stable remnant tumor) with substantially unchanged clinical status (**Table 1**).

Otherwise, regarding anaplastic PXA (7 cases), only 2 patients (28.6%) were neuroradiological stable (tumor free) presenting a clinical improvement measured by means of the KPS/LPS score. Three patients (42.9%) presented progression disease although Stupp protocol and are prosecuting with a chemotherapeutic approach with the possibility of performing a re-do surgery; finally, in 2 cases (28.6%), progression disease was so quick and clinically worsening leaving space only for palliative therapies (**Table 1**).

## Discussion

To our knowledge, this is the first report evaluating the advantages of fluorescein-guided surgery in PXA resection. Given that the contrast uptake at the preoperative T1-weighted MRI reflects the fluorescent staining pattern of the tumor (9, 15), we hypothesized that the use of the SF would have positively affected the intraoperative magnification of these neoplasms. Indeed, the use of SF could improve tumor visualization and, therefore, the entity of tumor removal (14): our observation was associated with contrast enhancement on preoperative MRI, which was present in almost all tumors of our series.

Ten patients enrolled in the study were scheduled for surgery with a previous planning of macroscopic resection but keeping in mind the philosophy of maximal safe resection: in this series, we obtained GTR in more than half of the patients (8/10, 80.0%); this data can be explained by the fact that many tumors were located in such eloquent regions in which a sub-total resection is sometimes advisable, in order to avoid permanent neurological deficits. As a matter of fact, minimal residual tumor (lower than 10% of preoperative tumor volume) at postoperative MRI was expected in 4 cases out of the total 12 patients (33.3%), as it was involving eloquent areas (sensorimotor region, corticospinal tract, and arcuate fascicle) or due to the adherences to MCA insular or perforating branches and was therefore independent from the fluorescence visualization. Therefore, in these cases, the EOR was related to the identification of eloquent subcortical tissue during resection, by means of neurophysiological monitoring, that allowed obtaining a maximal safe resection. In fact, in all cases, surgeon-reported visibility of pathologic tissue was clearly enhanced by SF and YELLOW 560 filter view and almost always judged helpful for complete tumor resection.

Pleomorphic xanthoastrocytoma is an astrocytic tumor thought to originate from subpial astrocytes or their precursors (1, 7). It is a rare entity accounting for < 1% and it is mostly seen in children and adolescents, with a median age of onset of 22 years old (22). They can arise anywhere along the neuroaxis, but they usually originate in the temporal lobe inducing epilepsy as a presenting symptom. PXA is a relatively recent entity with variable clinical outcome and still current dilemma regarding the optimum treatment (1); the rarity of this disease precludes conducting a strong trial: currently, aggressive surgical resection is the more effective therapeutic intervention because of its irrefutable correlation with both overall survival (OS) and progression-free survival (PFS). The role of supramaximal resection is discussed but no evidence is present.

According to WHO CNS5 classification (3), pleomorphic astrocytomas are considered as circumscribed astrocytic gliomas in consideration of their more solid growth pattern, as opposed to the strongly infiltrative behavior of diffuse astrocytomas; they are include in the same group of the more frequent pilocytic astrocytomas (PA), though, at the same time and as discussed above, PXA present some infiltrative features which make them like adult-type diffuse astrocytoma. In relation to mitotic index and specific mutations, PXA are classified as a grade II/2 or III/3 tumors. Pediatric PXA patients have improved survival outcomes compared to their adult counterparts. PXA have a higher tendency to recur than other gliomas; indeed, PFS is similar between grade II/2 and grade III/3 tumors, whereas anaplastic pleomorphic xanthoastrocytomas, having a more aggressive behavior, present a worse prognosis with a significative impact on OS. Therefore, the WHO grading is still important, remaining a strong predictor of overall survival.

Few recent published series regarding this argument show that GTR was achievable in about half of the patients operated with white-light illumination (7, 22, 28). In this perspective, the application of new technical tools aiming at improving the EOR could be beneficial in the management of PXA. During the last years SF has emerged as intraoperative tracer able to improve brain-tumor visualization (14–16), due to its vascular, non-specific mechanism of action related to the accumulation in brain regions with BBB disruption (9, 12): these areas present an altered permeability that seems to correlate consistently with the contrast enhanced portions of T1-weighted MRI sequences, accounting for the staining capacity of this fluorescent tracer with tumors that uptake contrast (21). Previous experiences had suggested that the use of SF could be associated with a bright fluorescence of the tumor area in primary and recurrent high-grade gliomas (14, 15, 20, 29), in brain metastases (17), in primary CNS lymphomas (18), in gangliogliomas (16), and recently in the similar entity of circumscribed astrocytic gliomas which are the pilocytic astrocytomas (30): this was also associated with good results in term of extent of resection as well as PFS and OS. In particular, in the heterogeneous group of PA, a maximal safe resection was reached in more than 80% of cases. Use of the fluorophore 5-aminolevulinic acid (5-ALA), a biochemical precursor of hemoglobin that provokes the synthesis and accumulation of fluorescent porphyrins in different tumors, has been reported but with inconsistent results: in a recent systematic review by Schwake et al. (31), 5-ALA was evaluated in different pediatric tumors, including 3 PXA, and strong fluorescence enhancement could be detected in 1 case (32), therefore accounting for a judgment of usefulness only in the 33.3% of the entire series. No comparative study for this specific tumoral histotype are available in the current literature.

In addition, the recent introduction of a dedicated filter integrated on the surgical microscope, specific for the excitation and emission wavelength of sodium fluorescein, has further improved the intraoperative visualization of the tumor and the surrounding brain parenchyma (11, 12). This technological adjunct significantly affected the development of fluorescein-guided surgery having increased the discrimination of tumoral tissue from the surrounding viable brain parenchyma. Our results seem to suggest that fluorescein-guidance for surgical resection may improve the intraoperative discrimination of the tumor margin and potentially increase the GTR rate. As stated by Xue et al. (33), we want to underline that our opinion is that only the combination of the largest number of tools, also including fluorophores, can guarantee an optimal surgical radicality in CNS surgery.

Although intraoperative evaluation of the fluorescence was subjective in this study, the report of helpful fluorescence in all cases, particularly at the tumor margins, taken together with the rate of GTR, suggests a reproducible effect (**Figure 1**). Fluorescein is still undergoing feasibility tests, especially in combination with the specific YELLOW 560 filter. It is important to stress that, in most of the Countries, the use of SF as a tracer in neuro-oncology should still be considered off-label; thus, a widespread utilization of fluorescence-guided surgery will depend on the definitive approval by the competent authorities (12, 15). Optimal dosage and timing of SF in PXA surgery is neither known nor experienced in this study: also in this cohort of tumors, we have used the low dosage of 5mg/kg, intravenously injected at the time of patient intubation (11), as originally proposed by our group in January 2012 (20).

The main limitation of the presented study is represented by the lack of data about overall survival in grade II/2 tumors: given the rarity of PXA, a long-term follow-up is not available in our data; for this reason, along with the inclusion of both anaplastic and not PXA, a proper patient evaluation from a prognostic perspective was not possible. Another selection bias in addition to different tumor grading inclusion, is the association in a single cohort of both adult and pediatric cases. Moreover, we did not make any comparison between the use of SF and white-light illumination or other available fluorophores, like 5-ALA. The current state, it is difficult to compare these two important fluorophores since the different mode of action, administration and visualization (34). Despite these weaknesses, our study could represent a proof of concept that may indicate that fluorescein utilization is potentially useful in the identification of tumoral tissue and in achieving a high rate of GTR of PXA. As previously demonstrated with other lesions, further studies could better elucidate the contribution of fluorescein-guided technique in improving the intraoperative visualization during surgical resection of this tumor subtype. Furthermore, the effect of fluorescein on PFS and OS needs to be evaluated in a randomized, controlled clinical trial with adequate power to precisely assess the outcomes within a predefined observation period.

## Conclusion

Our data suggested a positive effect of fluorescein-guided surgery during resection of PXA with contrast enhancement on preoperative MRI. We assert that SF is a safe and feasible tool: the use of fluorescein and YELLOW 560 filter is a readily available method for fluorescence-guided tumor resection, allowing intraoperative tumor visualization similarly to contrast enhancement in brain MRI.

## Data availability statement

The raw data supporting the conclusions of this article will be made available by the authors, without undue reservation.

## Ethics statement

The studies involving human participants were reviewed and approved by the Ethical Committee of the Fondazione IRCCS Istituto Neurologico Carlo Besta. Written informed consent to participate in this study as well as for the publication of any potentially identifiable images or data included in this article was obtained from the individual(s), and minor(s)’ legal guardian/next of kin.

## Author contributions

JF, MB and FA: study concept and design. JF, ER and FA: critical revision of the manuscript for intellectual content. All authors: acquisition of data, data analysis and interpretation. PF and FA: study supervision. All authors contributed to the article and approved the submitted version.

## Funding

This study was partially supported by Associazione Paolo Zorzi per le Neuroscienze, ONLUS and by the Italian Ministry of Health (RRC).

## Conflict of interest

Francesco Acerbi received honoraria from the Carl Zeiss Meditec Company for lectures at International Congresses.

The remaining authors declare that the research was conducted in the absence of any commercial or financial relationship that could be constructed as a potential conflict of interest.

## Publisher’s note

All claims expressed in this article are solely those of the authors and do not necessarily represent those of their affiliated organizations, or those of the publisher, the editors and the reviewers. Any product that may be evaluated in this article, or claim that may be made by its manufacturer, is not guaranteed or endorsed by the publisher.
